# Pathogenic Microorganisms Linked to Fresh Fruits and Juices Purchased at Low-Cost Markets in Ecuador, Potential Carriers of Antibiotic Resistance

**DOI:** 10.3390/antibiotics12020236

**Published:** 2023-01-22

**Authors:** Gabriela N. Tenea, Pamela Reyes, Diana Molina, Clara Ortega

**Affiliations:** Biofood and Nutraceutics Research and Development Group, Faculty of Engineering in Agricultural and Environmental Sciences, Universidad Técnica del Norte, Av. 17 de Julio s-21 y José María Córdova, Ibarra 100150, Ecuador

**Keywords:** antibiotic resistance, multidrug resistant bacteria, fresh agriculture products, fruits, juices, retail markets, strawberries, gooseberries

## Abstract

The pathogenic microorganisms linked to fresh fruits and juices sold out in retail low-cost markets raise safety concerns as they may carry multidrug-resistant (MDR) genes. To evaluate the microbiological quality and safety of highly consumed fruits and derivatives in Imbabura Province, Ecuador, ready-to-eat strawberries (5 independent batches; *n* = 300 samples), and gooseberries (5 separate batches; *n* = 500 samples), purchased from a local fruit farm grower and low-cost retail market, along with 20 different natural fruit- and vegetables-based juices (3 independent batches; *n* = 60 samples) purchased from food courts located within the low-cost markets were analyzed. Bacteriological analysis showed that the microbial quality was lower as several indicators (*n* = 984) consisting of total coliforms (TCOL), total aerobes (AEROB), *Enterobacter* spp. (ENT), *Shigella* spp., (SHIGA), yeasts (YE), and molds (M) were detected. *Staphylococcus* spp. (STAPHY) was found in both fruits regardless of origin, while *Escherichia coli* (EC) isolates were found in strawberries but not gooseberries. *Salmonella* spp. (SALM) were detected in juices only. Antibiotic susceptibility testing showed multidrug resistance of several isolates. The hemolytic pattern revealed that 88.89% of EC and 61.11% of ENT isolates were beta-hemolytic. All STAPHY isolates were beta-hemolytic while SALM and SHIGA were alpha-hemolytic. Plasmid curing assay of MDR isolates (ENT, EC, SALM, and STAPHY) showed that the antibiotic resistance (AR) was highly indicative of being plasmid-borne. These results raise concerns about the consumption of MDR bacteria. However, good agricultural and industrial practices, behavioral change communication, and awareness-raising programs are necessary for all stakeholders along the food production and consumption supply chain.

## 1. Introduction

Cape gooseberry or uvilla, uchuva, aguaymantu (*Physalis peruviana* L.) is one of the main exotic fruits demanded in the world market [1]. Strawberries (*Fragaria* spp. x ananassa) are the most consumed berry crops and are valued for their flavor, aroma, and nutritional quality. However, these fruits have physical and microbial spoilage problems causing losses of up to thirty percent during the post-harvest stage and market storage [2,3]. Fruit contamination, inadequate postharvest handling and hygiene, excessive use of fertilizers and pesticides, and climate change are problems that promote a high risk of foodborne diseases, economic loss, and food insecurity [4]. The latter affects around 815 million people in the world, challenging the 2030 Agenda for Sustainable Development and the UN Decade of Action on Nutrition (2016–2025) [5]. However, there is an increase in the health problems of consumers due to the presence of enteropathogenic bacteria, and the fruits that are consumed with the skin (i.e., strawberries, apples, blackberries, and pears, among others) are the ones that present greater possibilities of becoming a vehicle of these bacteria [6]. 

Foodborne outbreaks linked to the consumption of fresh fruits and vegetables have been reported [7]. A recent research study performed in Ethiopia indicated the presence of several pathogens including *Staphylococcus* spp., *Salmonella* spp., and *Shigella* spp. in juices purchased from the local market [8]; thus, they are the cause of health problems. 

In Ecuador, low-cost selling facilities within the local market lack basic infrastructure, and fruits and vegetables are inappropriately stored, thus the sanitary quality of these sites can be compromised, becoming a public health hazard. The current legislation related to food quality indicated that the products intended to be sold in the market should be free of contaminants [9]. However, the Ministry of Public Health has reported several human pathologies related to food contamination [10]. Despite of the implementation of normative practices for food production and manipulation, there is no control or inspection at the selling sites. Depending on the raw materials, processing conditions, distribution, and consumption, various types of foods offer a wide variety of scenarios in which food poisoning, pathogens, or spoilage have the possibility of proliferating. The National Institute of Statistics and Censuses (INEC) indicates that gastrointestinal diseases were among the five main causes of morbidity in the total population [11]. Within the country, the province of Imbabura represents 40% of the total production of gooseberries at the national level, with production between 104 to 350 tons/ ha [12]. The Ministry of Agriculture, Livestock, Aquaculture, and Fisheries [13] reveals that the cantons of Otavalo, Cotacachi, and Ibarra have a high potential for the cultivation of gooseberries with 10,009.68 (ha) represents 41.18% of the total area. In addition, Ecuador produces around 30,000 tons/ha of strawberries annually, of which 60% are destined for national consumption, and the rest are exported [14]. Their consumption has increased considerably since consumers highlight their high nutritional content and buy this product looking for a pleasant sensory experience [15]. On the other hand, foods are a dynamic environment for the continuing transfer of AR determinants between bacteria [16,17]. Although animal-derived food products are considered the primary source of the spread of AR, fresh exotic fruits, and their derivatives, such as juices, remain an ongoing concern. Strawberries and gooseberries are sensitive to contamination by microorganisms as they are collected from open fields and not washed before distribution for retail; from the farm harvest suppliers to the selling points, they might present some physiological or mechanical damage, thus by the time they are sold in low-cost markets they might be already contaminated. Additionally, the inappropriate storage of fruits in paint buckets and the template environment may increase the chance of contamination of these raw resources. Additionally, because these fruits are used to prepare juices, there is a great possibility that the final products may be compromised. Furthermore, these final products have not been examined from a microbiological standpoint and are offered on the market unrestrictedly without any type of permission as nutritional drinks and treatments for various illnesses, according to the seller’s claims.

Knowing the potential sources of on-farm contamination of fresh fruits via irrigation water, human handlers, domestic animals, and biological soil amendments, in this study, we propose to evaluate the microbiological quality of ready-to-eat strawberries and gooseberries purchased from a local fruit farm grower (SFS, PFS) at the 5–6 stage of maturity in comparison with fruits at the same stage sold in the low-cost open-air marketplaces (SFM, PFM). In addition, we evaluated the quality of twenty different natural juices (containing fruits only, the mix of fruits with vegetables, or fruits with other ingredients of animal origin) sold in food courts within local low-cost markets or parks. Moreover, the antimicrobial profile of the selected bioindicator bacteria detected in each sample was assessed. Furthermore, the origin of the AR and hemolytic activity were assessed. This preliminary study might help local authorities to achieve a better understanding of what may happen during the fruit distribution from a farm grower to the market and to take further decisions to help both farmers and sellers for further consumer protection from developing diseases associated with the consumption of contaminated products with MDR pathogens. 

## 2. Results and Discussion

### 2.1. Prevalence of Microbial Indicators in Ready-to-Eat Strawberries and Gooseberries Sold in Retail Low-Cost Markets and Farm Growers

**Figure 1:**
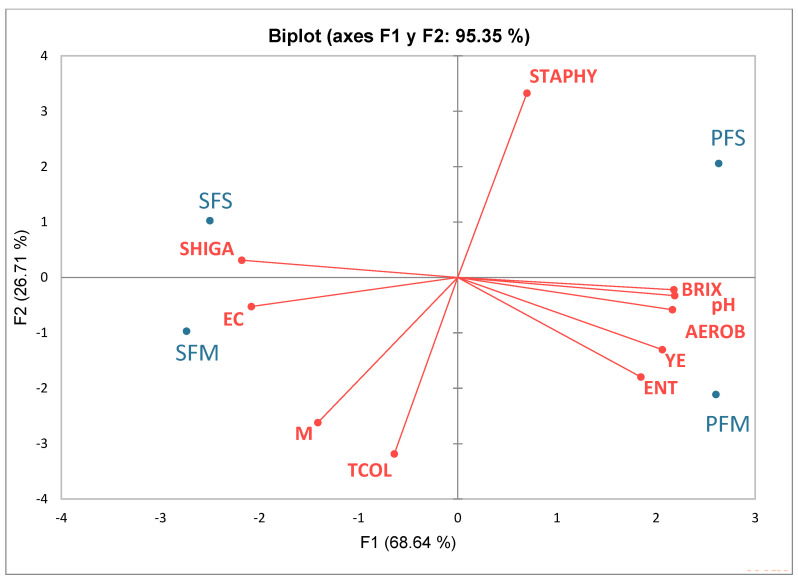
Biplot PCA analysis of 10 variables (pH, °Brix, TCOL, EC, AEROB, STAPHY, SHIGA, ENT, YE, M) of fruits at the ripening 6 stage. Legend: TCOL: total coliforms; EC: *E. coli*., AEROB: total aerobes; STAPHY: *Staphylococcus* spp., SHIGA: *Shigella* spp., ENT: *Enterobacter* spp., YE: yeasts; M: molds; SFS-strawberries from a local farm grower; SFM: strawberries from low-cost market; PFS: gooseberries from a local farm grower; PFM: gooseberries from low-cost market. Red color: active variables; Blue color: active observations.

The results of the bacteriological analysis showed the presence of various indicator bacteria in both ready-to-eat strawberries and gooseberries that were purchased from low-cost marketplaces and farm growers (Table 1). The study revealed that the quantity of TCOL was comparable in SFS (3.61 ± 0.18 logCFU/g) and SFM (3.66 ± 0.16 logCFU/g) fruits, despite the highest number of TCOL being found in PFM gooseberries purchased from the retail low-cost market (3.85 ± 0.37 logCFU/g). In addition, a comparable number of AEROBs have been detected in both fruits regardless of their origin of purchase. Results for the indicators ENT and STAPHY were unsatisfactory because they were detected in both fruits regardless of the source while SHIGA and EC were found in strawberries but not gooseberries. There were no SALM colonies found. All samples, regardless of their provenance, contained yeasts or molds (Table 1). Several harmful bacteria were associated with fruits and vegetables sold in open-air retail marketplaces [18]. Fruits and vegetables sold in wholesale markets and retail shops in Delhi-NCR were highly contaminated with potential human pathogens [19]. In addition, a recent metadata analysis of the published literature carried out in high- and low-income countries worldwide revealed the presence of pathogens in several raw fruits and vegetables during pre- and post-harvest periods [20]. Although no significant differences (*p* > 0.05) were obtained when compared among the groups, there was a high correlation between total solids (Brix), pH, and AEROB with a value of 0.993 and 0.986, respectively (data not shown). Even though both fruits had an acidic pH ranging from 3.33 to 3.78, we noticed that EC and SHIGA were solely absent from the gooseberries (Table 1). It is possible that EC in strawberries prevailed since those fruits are not cleaned after post-harvesting and gooseberries have the protective calyx. In addition, strawberries were found next to other vegetables in paint buckets, and people frequently handle those produce to assess their freshness or maturity, which could lead to contamination. In a prior study, it was demonstrated that *E. coli* O157:H7 may survive during production and post-harvest handling on the strawberry surface and inside the pulp [21]. The removal of the calyx from cape gooseberries induces an increase in the respiration rate, enhancing the level of ethylene and accelerating their decay [3]. Although the fruits were bought with calix, in this study, a greater quantity of TCOL, YE, and M were found in the PFM than in the PFS. The variation in fruit origin and their physiological characteristics after harvest may be connected to some pathogenic bacteria’s high capacity to adapt and colonize the raw material to ensure their metabolic activities. A PCA analysis of both fruits and 10 variables (pH, Brix, TCOL, AEROB, EC, SHIGA, ENT, STAPHY, YE, and M) revealed a distinct difference between fruits produced on farms and those sold in the low-cost markets (Figure 1). The F1 component explained 60.10% of the total variance being loaded in the positive (+) direction with pH, Brix, STAPHY, ENT, YE, and AEROB, while the F2 explained 29.42% of the variance being loaded in the negative (−) direction, with SHIGA, TCOL, EC, and M (Figure 1). The pH, Brix, and SHIGA are those that most influence the main component or axis F1 (16.40%, 16.30%, and 16.29%) while the TCOL and STAPHY vectors contribute 22.96%, and 31.37%, respectively, to the main component F2. We observed that the variables pH and Brix were close together, indicating a high correlation between these cutoff values throughout the experiment. Based on the results, SFS and PFS showed high STAPHY content, whereas SFM showed the high content of molds, and PFM had a high content of ENT. The product must meet the microbiological standards established in accordance with the Principles for the Establishment and Application of Microbiological Criteria for Foods (CAC/GL21-1997, IDT) [22]. Based on these results, the analyzed samples were unsatisfactory as the total indicator microorganisms counts except SALM were above the accepted limit [9]. Overall, our analysis agreed with previous studies [8,18], linking the contamination of fruits during post-harvest inappropriate manipulation, transporting, packaging, food handlers’ hygiene, and storage conditions, the level of contamination and the presence of a certain indicator might depend on the raw material composition and their physicochemical characteristics. Further safety measurements need to be taken after fruits post-harvest before being distributed to the retail market. 

### 2.2. Prevalence of Microbial Indicators among Natural Juices Expanded in the Low-Cost Courts 

**Figure 2:**
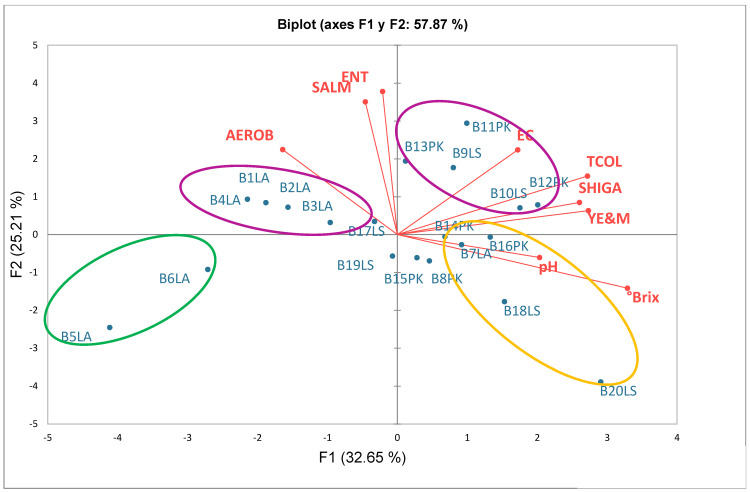
Biplot PCA analysis of 9 variables (pH, °Brix, TCOL, EC, AEROB, SALM, SHIGA, ENT, YE, M) of juices (20) purchased from the food courts. Legend: TCOL: total coliforms; EC: *E. coli*., AEROB: total aerobes; SALM: *Salmonella* spp., SHIGA: *Shigella* spp., ENT: *Enterobacter* spp., YE&M: Yeasts/Molds. LA: low-cost market Amazonas; PK: park Centrica; LS: low-cost market Santo Domingo. The color circles marked the close related samples. Red color: active variables; Blue color: active observations.

Due to the richness of exotic fruits and vegetables, the Ecuadorian market provides a wide variety of juices. The prevalence of common harmful germs in these products was not investigated. According to data gathered from a complementary survey response regarding the consumption of juice and their motivation, 77.45% of the interviewed local respondents (49 women and 53 men) purchased these juices from low-cost markets (Supplementary Materials Figure S1A) and more than 66% consumed those juices to treat or prevent various disorders (Figure S1B). Thus, based on this survey, we concluded that juice consumption is mainly related to various health benefits; nonetheless, respondents were aware of the risks associated with the presence of pathogens in these drinks and how they can increase the risk of foodborne infections. However, among the 20 analyzed fruit- and vegetable-based juices, the bacteriological results indicated that TCOL and AEROB content varied among samples (Table 2). Nonetheless, 6 samples (30%) tested positive for all indicators except for STAPHY, which was not found in any juice samples. According to the findings, 50% of the juice samples contain the indicator EC while 90% of the samples included ENT. In 80% of the samples, SHIGA and SALM were found. The Ecuadorian Institute of Normalization regarding the microbiological specifications of unpasteurized products does not contemplate the allowed number of total coliforms, *E.coli*, *Salmonella*, *Staphylococcus*, yeasts, and molds, except in the pasteurized products [23]. Instead, under European law, juices like smoothies must pass a detection threshold [24]. However, *E. coli* is prohibited in unpasteurized fruits and vegetable juices (ready-to-eat) following Commission Regulation (EC) No. 2073/2005 [24]. Leafy greens including spinach, lettuce, parsley, and cilantro have a higher prevalence of *Salmonella* than other varieties of non-leafy green vegetables, according to an earlier study [25,26]. Coliform bacteria and enterococci have mostly been utilized as indicators of postprocessing contamination in foods [27]. However, their presence may indicate serious carelessness in the handling of natural juice ingredients. Recently, it was observed that *E. coli* and fecal coliforms were more common in fresh vegetables in Thailand [18]. Thus, fresh products from open-air markets had greater fecal coliform and *E. coli* concentrations than produce from grocery stores. *Shigella* and *Salmonella* prevalence was 4.8% and 0.4%, respectively. In the present study, only 30% of the samples were yeast- and mold-free, with the B10 sample having the highest yeast content (4.26 ± 0.15 logCFU/mL) (Table 2). It is challenging to determine from these analyses whether there is a relationship between the juice composition (Table S1), physicochemical condition, and the presence of a specific pathogen, considering that the samples are divergent in their composition, pH, and Brix. Nonetheless, a biplot using the PCA scores and factor loading equates the similarities of the variables obtained (Figure 2). The variable F1 explained 32.65% of the total variance while F2 explained 25.21%. Based on the results, we observed that pH and Brix were factors that influenced the clustering (yellow circle) of the samples collected from both park (PK) and the low-cost market Santo Domingo (LS). The other samples were clustered based on the indicator type (magenta circle). A cluster was observed with the juices originating from the low-cost market Amazonas (LA) that were loaded with the vector AEROB. Samples B5 and B6 were located distant from the pH and Brix vectors, meaning that they were divergent in pathogens content and physicochemical characteristics (green circle). However, a Pearson correlation coefficient test was performed to study in detail the intensity of the correlations between the variables (Table 3). A moderate correlation was seen in the pH and Brix variables with a value of 0.576. In addition, the variables Brix, SHIGA, and YE&M showed a moderate correlation with the values 0.446 and 0.467, respectively. In addition, a moderate correlation was observed between TCOL and YE&M with a value of 0.664 and SALM and ENT with a value of 0.564. A moderate inverse correlation between Brix and AEROB content was also found with a value of -0.486. This suggests that at high Brix, AEROB declined; however, Pearson’s correlation does not always support this conclusion. In a recent study regarding the prevalence of pathogenic bacteria in juices collected from the Ethiopian market, the bacteriological analysis indicated that 74.4% of samples were contaminated with *Staphylococcus* while *Salmonella* and *Shigella* were detected in 24.4% and 15.4% respectively [8]. In another study, among 20 smoothie drinks prepared from fruits and vegetables collected from 6 food services in Slovakia, antibiotic-resistant coliforms prevailed in at least one of each food service [27]. It is crucial to store and handle raw materials (fruits) carefully because they are highly perishable. Taken together, these results indicated that natural ready-to-drink juices expanded in the local market do not comply with the microbiological requirements. According to national legislation, the product must be free of pathogenic bacteria, toxins, and any other microorganism that causes the decomposition of the product [23]. In addition, the product must be free of any substance originated from microorganisms and that represents a risk to health. Nonetheless, we suggest that the safety of these products is at risk due to inappropriate preparation and improper handling of raw material and final products (handling money and preparing the juice while not wearing safety gloves, etc.). Thus, rather than offering healthy products as claimed, they offer products with a high content of pathogenic bacteria that can raise the risk of foodborne infections of the customers. The municipal authorities should also consider product inspection and quality assurance. The staff must therefore obtain training in food safety to decrease the likelihood of hazardous microorganisms in these foods. 

### 2.3. AR Profile 

**Figure 3:**
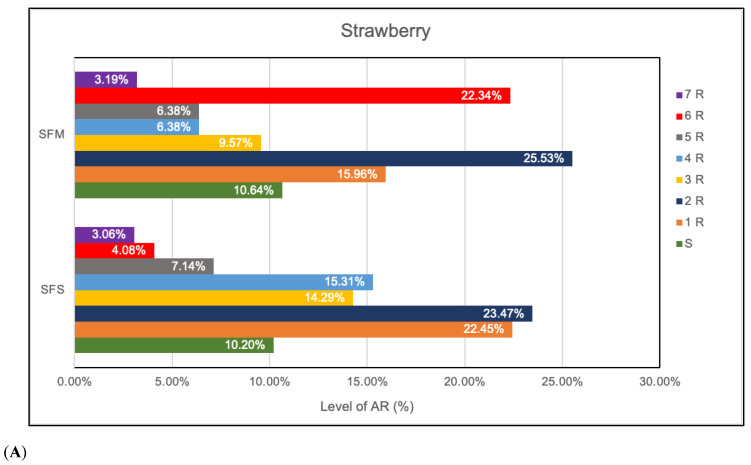

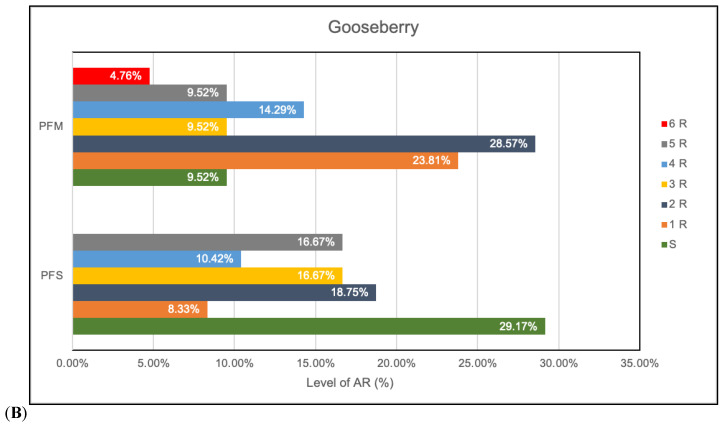
Level of AR (%) in (**A**). strawberry; (**B**). gooseberry. The bars represent the % of bacteria that showed resistance to different number of antibiotics tested. Legend: SFS-strawberries from a local farm grower; SFM: strawberries from low-cost market; PFS: gooseberries from a local farm grower; PFM: gooseberries from low-cost market; S- susceptible; R-resistant.

Fresh produce that has been eaten raw may include antimicrobial residues, microbes that are resistant to antibiotics, and clinically important antimicrobial resistance genes [20]. A detrimental effect on human health was demonstrated by the rise of AR linked to microorganisms in food [28]. An early study indicated that bacteria detected in fruits and vegetables were the most frequently seen antibiotic-resistant [29]. The AR profiles of the colonies selected from SFS and SFM strawberries in this investigation were different, with 15.31% of SFS samples being resistant to four antibiotics while 22.34% of SFM samples were resistant to six antibiotics (Figure 3A); 3.19% of the samples exhibited resistance to 7 antibiotics. According to the findings, the selected isolates were resistant to at least one or two antibiotics from various classes (cephalosporins, beta-lactamases, aminoglycosides, and penicillin-like antibiotics) (Table S2). STAPHY isolates from both SFS and SMF were 100% resistant to vancomycin and methicillin. Additionally, 44% and 25% of SMF samples were resistant to gentamycin and cefuroxime, respectively, while all selected isolates from SFS fruits were sensitive to both drugs (Table S2). Likewise, the antibiotic profile varied between PFS and PFM fruits, 4.76% of the chosen colonies displayed 6AR in the PFM stage (Figure 3B). The PFS stage showed the largest percentage of 5AR (16.67%),while the PFM stage showed the highest percentage of 4AR (Figure 3B). STAPHY isolates were resistant to methicillin (100%) while ENT isolates were resistant to all antibiotics except tetracycline among the indicators found in both PFS and PFM (Table S3). It is concerning that foods may contain methicillin-resistant *Staphylococcus*, and this should not be disregarded [30]. A previous study indicated the presence of *S. aureus* in food handler samples (53.3%) from Brazil State public schools [31]. The results revealed that to ensure sufficient sanitation, the food handlers needed to reevaluate their food safety training. In addition, this bacterial group has been identified in both meat and dairy products, including milk and cheese [31,32]. According to Gutiérrez et al. [33], the food supply chain serves as a shuttle for the spread of resistant bacteria to people. 

**Figure 4:**
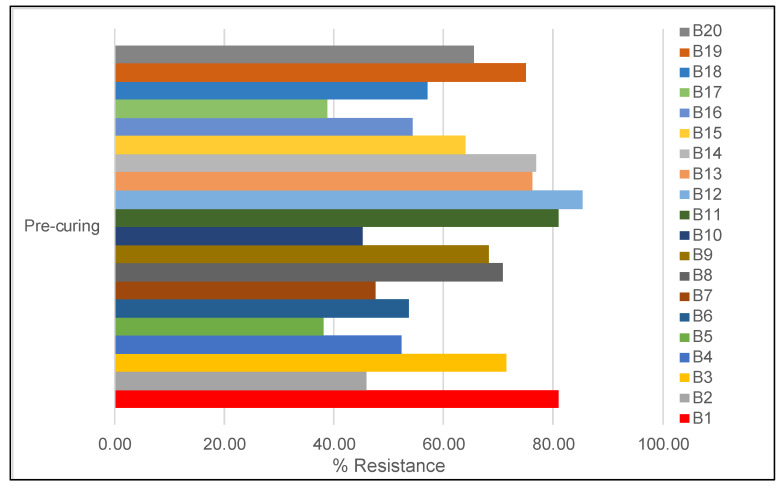
Level of AR (%) in juices. The bars represent the % of bacteria that showed resistance.

A different level of AR was also detected among juices purchased from low-cost marketplaces. Figure 4 shows the prevalence of AR with 15 juices displaying more than 50% resistance. The juices B1, B11, and B12 showed the strongest resistance (>80%), whereas juices B5 and B17 showed the lowest resistance (>38%). The percentage of resistance for each category of the indicator is shown in Table S4. The indicator EC detected in the B16 sample was sensible to all antibiotics except for amoxicillin. SHIGA, ENT, and SALM displayed different degrees of AR; this might be related to the raw matrix source. Based on these findings, we noticed that almost all juice isolates were resistant to at least three of the antimicrobials examined, indicating a high level of competitiveness in the environment. However, improper handling and inadequate hygiene standards at retail markets could also be contributing factors. Additionally, the fruits will probably become contaminated with MDR bacteria given that the farm fruits originated from a field where the farmers are using animal excrements as fertilizer; this should be further investigated. Therefore, the consumption of raw fruits and vegetables contaminated with MDR bacteria may increase the risk of AR genes spreading by horizontal gene transfer within the gut. This findings are consistent with recent review conducted on fresh produce contaminated with antibiotic resistant bacteria [20]. At this point, based on these results it is not possible to determine the extent of the human health risk associated with the consumption of these products containing AR bacteria; further analyses for risk assessment purposes are required. 

### 2.4. Hemolytic Phenotype of Selected MDR Isolates

Numerous bacterial species produce hemolysins, which are regarded as a crucial element of pathogenicity. To provide the toxin-producing bacteria with nutrients, especially iron, these chemicals break membranes, cause cells to lyse, and kill adjacent cells and tissues [34]. In this study, a considerable number of isolates associated with fruits and juices were beta-hemolytic according to the transparent zone to surround bacterial colonies on blood agar (Table 4). In the human blood agar-containing media, beta-hemolytic activity was found in all STAPHY isolates from both fruits. While 95.83% of SHIGA were alpha-hemolytic, only 88.89% and 66.11% of the EC and ENT isolates from strawberries were beta-hemolytic. A significant portion of the EC (76.87%) and ENT (82.55%) isolates linked to juices were found to be beta-hemolytic. Juice-isolated SALM and SHIGA were not beta-hemolytic. Early studies associate beta-hemolytic *E. coli* with human infections [35]. In addition, a frequent pathogen that causes both hospital- and community-acquired illnesses is *Staphylococcus aureus* [36]. Hemolysin, one of the key virulence factors for *S. aureus*, induces the usual beta-hemolytic phenotype, also known as the complete hemolytic phenotype [35]. Nonetheless, further analysis to detect the virulence factors is required for better characterization of isolates.

### 2.5. Origin of AR 

**Figure 5:**
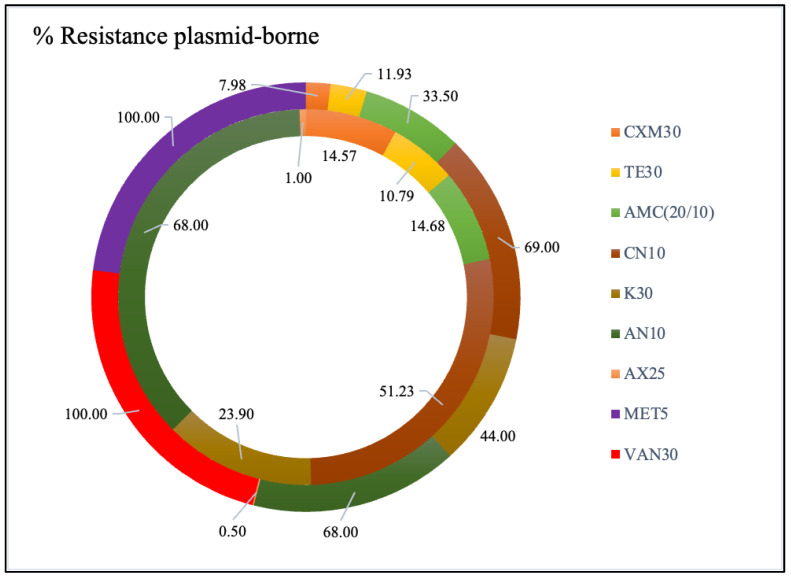
Plasmid-borne antibiotic profile of MDR isolates from fruits (outer circle) and juices (inner circle). Legend: K30: kanamycin 30 (μg); CN10: gentamycin 10 (μg); AN10: ampicillin 10 (μg); AX25: amoxicillin 25 (μg); TE30: tetracycline 30 (μg); CXM: cefuroxime 30 (μg); VAN30: vancomycin 30 (μg); MET5: methicillin 5 (μg); AMC: amoxicillin: clavulanic acid (20/10 μg).

Genes for AR, catabolic processes including lactose utilization and hydrocarbon breakdown, and the production of specific antibiotics are all found on bacterial plasmids [37]. To determine what causes AR, it is possible to cure (remove) plasmids from several bacterial strains. The superhelical structure of the plasmid DNA would then be broken by intercalating agents, such as ethidium bromide, resulting in the production of an open circular or linear form of plasmid DNA [38]. When healed plasmids have no impact on resistance, it is often classed as “plasmidic,” and when they do, it is categorized as “chromosomal.” The isolates exhibiting AR were indicative of being plasmid-borne (Figure 5). When tested against AN10, the results showed that 68% of the isolates from both fruits and juices were plasmid-borne, compared to 51.25 to 69% when tested against CN10. Additionally, when examined for resistance to MET5 and VAN30, the resistant STAPHY isolated from fruits was 100% of plasmid origin. Previous research indicates the resistance to methicillin of staphylococci associated with food [39]. In addition, several pathogens detected in fresh fruits and vegetables were highly resistant to several antibiotics, this resistance being plasmid related [39]. Our understanding of the transmission of plasmid-mediated AR may be improved by further categorizing the plasmids and studying their distribution and evolution in different bacterial hosts.

## 3. Materials and Methods

### 3.1. Samples Selection and Processing 

The fruit samples were purchased from a local farm grower and low-cost open market while the fruit-based juices were purchased from the food courts located within the market or street (Table S1). Samples consisted of (1) strawberry fruits of ripening stage 5–6 (SFS) from a local farm grower (*n* = 150), (2) strawberry fruits stage 5–6 (SFM) purchased from the low-cost market (*n* = 150), (3) gooseberries of ripening stage 6 (PRS) purchased form a local farm grower with calyx (*n* = 250), and (4) gooseberries from the low-cost market at stage 6 (PRM) with calyx (*n* = 250); 5 independent batches were purchased at the one-month interval. In the case of a farm grower, the owner was asked to collect randomly healthy fruits from the field without any visible damage. This study was realized between January–October 2022, regardless of the season. In addition, 20 different fruit- and vegetable-based juices (200mL of each 3 batches/ juice; *n* = 60 samples) were purchased in plastic bags or plastic recipients as they were sold by local vendors in the food courts of low-cost markets (LA: Amazonas: 6 juices; LS: Santo Domingo: 7 juices) and parks (PK: Centrica: 7 juices). The juices were prepared freshly by using a blender. In the case of the mixed juices, the ingredients of animal origin were kept in plastic boxes and added to the juice according to the vender recipe. The recollection of juice samples was realized between June–November 2019 and February–July 2020, regardless of the season, and was chosen as the stands are permanently located in the market. Table S1 described the juice composition and health benefits according to the retailer description. All samples were brought to the laboratory and immediately proceeded to bacteriological analysis. 

### 3.2. Bacteriological Analysis 

The microbiological quality was performed as previously described [40]. Briefly, 25 g of chopped fruits, randomly collected from the initial 500 g of field fruits or fruits purchased from low-cost marketplace, were placed in a zip bag containing buffered peptone water (0.1%) and mixed vigorously by hand for the 30 s. Moreover, the liquid was poured in a centrifuge bottle and incubated at 37 °C for 4 h. Similarly, 25 mL of each juice sample was used. The presence of total coliforms (TCOL), total aerobes (AEROB), *E. coli* (EC), *Salmonella* spp. (SALM), *Shigella* spp. (SHIGA), *Enterobacter* spp. (ENT), *Staphylococcus* spp. (STAPHY), yeasts (YE), and molds (M) were assessed according to the Ecuadorian legislation [9,41]. Briefly, decimal dilutions made with sterile water were inoculated on 3M Petrifilm Aerobic (3M Science Applied to Life, Detroit, MI, USA), to determine the AEROB microbial population (37 °C, 48 h). For the detection and differentiation of the possible presence of SALM and SHIGA, aliquots (100 μL) were inoculated onto SS (Shigella-Salmonella SS Agar, Difco, Detroit, MI, USA) and incubated for 48 h at 37–40 °C. The presence of SALM was confirmed according to [42]. Separate experiment aliquots (100 μL) were placed on Chromocult Coliform agar (Merck, Rahway, NJ, USA) to determine the TCOL and EC, as well as eosin methylene blue (Difco, Detroit, MI, USA), to detect the presence of ENT and EC. Additionally, yeasts and molds were counted using 3M Petrifilm Yeast and Mold (3M Science Applied to Life, MI, USA) (7 days incubation at 25–28 °C). Furthermore, the presence of STAPHY was determined in Brilliance Staph 24 Agar Medium (Oxoid Limited, Wade Road, Basingstoke, Hampshire, UK) [43]. Microbial counts were expressed as CFU/(g, mL). At least ten colonies for each biological indicator (classified at the genus level) detected in the samples were randomly extracted and purified and then used for antibiotic susceptibility testing. 

### 3.3. Physicochemical Analysis 

The pH of all samples (fruits and juices) was measured using a pH meter (Seven Compact S210, Mettler Toledo LCC, Columbus, OH, USA). Total soluble solids (°Brix) of total sugar in the fruit were determined using a digital refractometer.

### 3.4. Antibiotic Susceptibility Testing

A total of 192 random colonies from the detected bioindicator isolates in strawberries (SFS: 98; SFM: 94), 80 random colonies selected from gooseberries (PRS:40; PRM: 40), and 712 colonies from the 20 different juices were picked up and used for antibiotic susceptibility test using commercial discs, such as amoxicillin (AMX25: 25 μg), ampicillin (AN10: 10 μg), gentamicin (CN10: 10 μg), kanamycin (K30: 30 μg), tetracycline (TE30: 30 μg), and cefuroxime (CXM30: 30 μg). In addition, selected isolates from fruits were tested for vancomycin (VAN:30 μg) while the selected isolated from juices were tested for amoxicillin/clavulanic Acid (AMC30: 20/10 μg). In addition, 50 STAPHY colonies randomly picked up from gooseberries and strawberries were tested for susceptibility to methicillin (MET5: 5 μg). For the disk diffusion assay, we used the concentrations recommended by the Scientific Committee on Animal Nutrition (discs provided by Merck, USA). The Scan500 (Interscience, Fr) was used to determine the inhibitory halos automatically and to categorize them according to the microbiological breakpoints reported by the FEEDAP standards, as susceptible, intermediary, or resistant [44]. As a reference, *E. coli* ATCC25922, *S. aureus* ATCC1026, *S. enterica* subsp. *enterica* ATCC51741, and *S. sonnei* ATCC23931 were used. The % of AR was determined as the number of total bacteria resistant/the number of total isolates tested. Moreover, the level of AR (%) was determined as number of isolates showing resistance to 1 and up to 8 antibiotics/total number of isolates.

### 3.5. Hemolysis Test 

The hemolytic activity of selected MDR isolates was determined on Columbia agar containing 5% (*w*/*v*) human blood [45]. After 48h of incubation at 37 °C, hemolytic activity was assessed, and the isolates were classified based on the lysis of red blood cells in the medium adjacent to colonies: green regions near colonies (alfa-hemolytic), clear region near colonies (beta-hemolytic), and no regions near colonies (gamma-hemolytic). 

### 3.6. “Plasmid Curing” Assay

Plasmid curing (elimination) test was performed to determine the location (plasmid-borne or chromosomal) of the AR marker (s) using sublethal concentrations of ethidium bromide as described by Bouanchaud et al. [46] with slight modifications. Briefly, a 24 h culture of selected ENT, EC, SALM, and STAPHY colonies showing resistance to at least four antibiotics (a total of 75 colonies randomly selected from fruits and juices) were inoculated into 5 mL of nutrient broth containing different concentrations of ethidium bromide (50, 75, and 125 μg/mL) and incubated at an appropriate temperature for 24 h [46,47,48]. After the incubation time, serial dilutions were prepared, and the cultures were plated onto prepared Müller Hilton agar plates and incubated at 37 °C for 2–3 h before the discs were placed on the surface of the plates (spot) as described in Section 3.4. The plates were incubated at 37 °C for 48 h, and the inhibition halo was determined. Absence of an inhibition zone on agar Mueller-Hinton indicates plasmid-mediated resistance (plasmid cured) while the presence of an inhibition zone indicates chromosome-mediated resistance (plasmid not cured).

### 3.7. Statistical Analysis 

All experiments were performed in triplicate. The results were reported as mean ± standard deviation. The normal distribution of the data was employed with the Shapiro-Wilk test [49]. The Fisher Least Significant Difference test was applied to determine significant differences between the means (SPSS 13.0, Inc., Chicago, IL, USA). Moreover, PCA analysis of 10 variables (pH, °Brix, TCOL, AEROB, EC, SHIGA, ENT, STAPHY, YE, and M) was conducted on fruits purchased from the farm growers and low-cost market. Similarly, PCA analysis was conducted on 9 variables (pH, °Brix, TCOL, AEROB, EC, SHIGA, ENT, SALM, YE&M) on the 20 juices on day of purchasing. In addition, a Pearson correlation was performed to find whether there is an interaction between the response variables.

## 4. Conclusions

This is the first report on microbial safety and prevalence of some bacterial indicators (EC, ENT, SALM, SHIGA, STAPHY) of exotic fruits and derivates (juices) expanded to low-cost (open-air) marketplaces in Ecuador. According to this study, ready-to-eat strawberries and gooseberries from retail market showed greater levels of contamination with pathogens, including *Staphylococcus* spp. and *Enterobacter* spp. along with a high content of yeasts and molds, thus the bacteriological safety of these products is compromised. In addition, several juices based on a mix of tropical fruits, vegetables, and other meat-based ingredients showed a high content of total coliforms, *Salmonella* spp., *Shigella* spp., *Enterobacter* spp., and *E. coli*. These indicative pathogens were found resistant to various antibiotics, and the resistance was detected as plasmid-borne. The presence of MDR bacteria in fresh fruits and natural juices consumed in Ecuadorian retail low-cost markets continues to be of concern; thus, there is a need to develop strategies to ensure food security and safety by lowering bacterial contamination in both farm and retail markets and to prevent or reduce the spread of antibiotic resistant infections within the population. 

## Figures and Tables

**Table 1:** Physicochemical characteristics and prevalence of microbial indicators in fresh strawberries and gooseberries.

Fruit	Sample Code	Total Solids (°Brix)	pH	Total Coliforms	Total Aerobes	*E. coli* spp.	*Enterobacter* spp.	*Staphylococcus* spp.	*Shigella* spp.	Yeasts	Molds
log CFU/ g											
Strawberry	SFS	9.02 ± 0.15	3.38 ± 0.37	3.61 ± 0.18	2.24 ± 0.34	1.99 ± 0.22	2.19 ± 0.22	2.61 ± 0.15	0.74 ± 0.19	0.89 ± 0.16	1.45 ± 0.18
	SFM	9.24 ± 0.29	3.53 ± 0.24	3.66 ± 0.16	2.35 ± 0.16	3.61 ± 0.34	2.17 ± 0.18	2.01 ± 0.37	0.59 ± 0.33	1.23 ± 0.18	1.99 ± 0.24
Gooseberry	PFS	12.91 ± 0.37	4.32 ± 0.15	3.08 ± 0.22	2.71± 0.17	(-)	2.48 ± 0.37	2.92 ± 0.15	(-)	3.08 ± 0.22	1.02 ± 0.25
	PFM	12.78 ± 0.22	3.38 ± 0.22	3.85 ± 0.37	2.76 ± 0.18	(-)	3.01 ± 0.24	2.11± 0.24	(-)	4.53 ± 0.37	1.57 ± 0.26

Data represent mean ± standard deviation of 3 experimental repetitions (-): not detected. Legend: SFS-strawberries from a local farm grower; SFM: strawberries from low-cost market; PFS: gooseberries from a local farm grower; PFM: gooseberries from low-cost market.

**Table 2:** Physicochemical characteristics and prevalence of microbial indicators in fresh juices.

Juice Code	°Brix	pH	Total Coliforms	Total Aerobes	*E. coli*	*Enterobacter* spp.	*Salmonella* spp.	*Shigella* spp.	Yeasts/Molds
log CFU/mL									
B1	4.01 ± 0.01	4.15 ± 0.01	3.01 ± 0.24	5.18 ± 0.15	(-)	2.78 ± 0.37	(-)	2.01 ± 0.24	(-)/(-)
B2	4.82 ± 0.01	3.89 ± 0.01	2.14 ± 0.15	2.85 ± 0.37	2.71 ± 0.24	3.09 ± 0.22	2.47 ± 0.37	1.41 ± 0.24	(-)/(-)
B3	7.91 ± 0.01	4.32 ± 0.01	3.25 ± 0.37	4.36 ± 0.15	(-)	3.05 ± 0.18	2.01 ± 0.37	1.91 ± 0.24	(-)/(-)
B4	4.02 ± 0.01	4.02 ± 0.01	1.02 ± 0.02	3.95 ± 0.37	2.59 ± 0.22	4.09 ± 0.22	1.69 ± 0.37	2.01 ± 0.33	(-)/(-)
B5	2.02 ± 0.01	2.05 ± 0.01	1.47 ± 0.38	3.91 ± 0.24	(-)	1.39 ± 0.22	1.89 ± 0.38	1.88 ± 0.34	(-)/(-)
B6	4.01 ± 0.01	5.01 ± 0.01	1.38 ± 0.02	4.94 ± 0.24	(-)	1.48 ± 0.38	2.08 ± 0.37	(-)	2.03 ± 0.33/ (-)
B7	9.81 ± 0.01	3.95 ± 0.01	3.31 ± 0.24	2.19 ± 0.22	3.01 ± 0.24	2.04 ± 0.24	1.91 ± 0.37	(-)	2.18 ± 0.17/(-)
B8	8.41 ± 0.01	4.81± 0.01	2.75 ± 0.37	2.69 ± 0.22	(-)	1.95 ± 0.37	1.53 ± 0.33	1.94 ± 0.33	1.54 ± 0.37/(-)
B9	7.51 ± 0.01	3.97 ± 0.01	3.89 ± 0.22	4.08 ± 0.38	(-)	3.06 ± 0.15	(-)	1.83 ± 0.33	3.63 ± 0.33/(-)
B10	8.91 ± 0.01	3.58 ± 0.01	4.49 ± 0.22	1.78 ± 0.38	2.13 ± 0.18	3.19 ± 0.38	1.95 ± 0.18	1.95 ± 0.18	4.26 ± 0.15/(-)
B11	7.01 ± 0.01	3.99 ± 0.01	4.29 ± 0.22	5.05 ± 0.24	3.95 ± 0.37	4.05 ± 0.18	2.26 ± 0.15	2.31 ± 0.38	3.45 ± 0.18/(-)
B12	12.2 ± 0.01	6.74 ± 0.01	2.78 ± 0.38	4.94 ± 0.15	(-)	2.53 ± 0.33	2.04 ± 0.33	(-)	3.17 ± 0.18/(-)
B13	8.11 ± 0.01	3.77 ± 0.01	3.38 ± 0.38	4.87 ± 0.38	(-)	3.89 ± 0.22	1.78 ± 0.37	1.54 ± 0.17	2.72 ± 0.33/(-)
B14	7.01 ± 0.01	4.32 ± 0.01	3.81 ± 0.24	2.81 ± 0.24	(-)	3.08 ± 0.38	1.48 ± 0.37	1.94 ± 0.17	3.81 ± 0.24/(-)
B15	8.71 ± 0.01	4.07 ± 0.01	2.95 ± 0.37	2.85 ± 0.15	(-)	2.94 ± 0.17	1.31 ± 0.24	1.95 ± 0.18	3.12 ± 0.22/(-)
B16	9.62 ± 0.01	4.01 ± 0.01	3.81 ± 0.24	2.93 ± 0.17	2.61 ± 0.24	2.81 ± 0.24	1.02 ± 0.22	2.04 ± 0.17	3.08 ± 0.18/(-)
B17	9.01 ± 0.01	4.56 ± 0.01	3.06 ± 0.15	3.69 ± 0.22	2.95 ± 0.37	2.73 ± 0.17	1.93 ± 0.33	1.45 ± 0.37	(-)/(-)
B18	11.31 ± 0.01	5.61± 0.01	3.05 ± 0.24	2.48 ± 0.02	2.31 ± 0.24	(-)	(-)	1.74 ± 0.24	2.31 ± 0.24/(-)
B19	11.61 ± 0.01	3.51 ± 0.01	3.30 ± 0.24	2.31 ± 0.24	2.01 ± 0.24	2.64 ± 0.24	1.78 ± 0.33	(-)	2.48 ± 0.37/(-)
B20	16.51 ± 0.01	5.62 ± 0.01	2.99 ± 0.37	2.01 ± 0.24	1.18 ± 0.15	(-)	(-)	2.81 ± 0.24	2.47 ± 0.18/(-)

Data represent mean ± standard deviation of minimum 3 experimental repetitions; (-): not detected.

**Table 3:** Pearson correlation coefficient of 9 variables.

Variables	°Brix	pH	TCOL	AEROB	EC	ENT	SALM	SHIGA	YE&M
°Brix	**1**	**0.576**	**0.457**	**−0.486**	0.299	−0.346	−0.314	**0.446**	**0.467**
pH	**0.576**	**1**	0.005	0.068	0.202	−0.255	−0.032	**0.458**	0.196
TCOL	**0.457**	0.005	**1**	−0.227	0.285	0.275	0.178	**0.469**	**0.664**
AEROB	**−0.486**	0.068	−0.227	**1**	0.050	0.323	0.415	−0.098	−0.246
EC	0.299	0.202	0.285	0.050	**1**	0.378	0.284	0.289	0.258
ENT	−0.346	−0.255	0.275	0.323	0.378	**1**	**0.564**	0.155	0.085
SALM	−0.314	−0.032	0.178	0.415	0.284	**0.564**	**1**	0.021	−0.008
SHIGA	**0.446**	**0.458**	**0.469**	−0.098	0.289	0.155	0.021	**1**	0.277
YE&M	**0.467**	0.196	**0.664**	−0.246	0.258	0.085	−0.008	0.277	**1**

Values in bold are different from 0 with a significance level alpha = 0.05. Legend: TCOL: total coliforms; AEROB: total aerobes; EC: *E. coli*; SALM: *Salmonella* spp.; SHIGA: *Shigella* spp.; ENT: *Enterobacter* spp.; YE&M: Yeasts/Molds.

**Table 4:** Hemolytic pattern (%) of the selected isolates originated from fruits and juices.

Samples	Selected Isolates	Hemolysis (%)		
		Beta	Alfa	Gamma
Strawberries	*E. coli* (*n* = 72)	88.89	11.11	0
	*Shigella* ssp. (*n* = 72)	4.17	95.83	0
	*Enterobacter* spp. (*n* = 90)	61.11	37.50	1.39
	*Staphylococcus* spp. (*n* = 40)	100.00	0.00	0
Gooseberries	*Enterobacter* spp. (*n* = 40)	62.50	37.50	0
	*Staphylococcus* spp. (*n* = 40)	100.00	0.00	0
Juices	*Enterobacter* spp. (*n* = 235)	82.55	17.45	0
	*E. coli* (*n* = 134)	76.87	23.13	0
	*Shigella* spp. (*n* = 194)	0.00	100.00	0
	*Salmonella* spp. (*n* = 149)	0.00	100.00	0

## Data Availability

Not applicable.

## Supplementary Materials

The following supporting information can be downloaded at: https://www.mdpi.com/article/10.3390/antibiotics12020236/s1. Figure S1. A. Poll survey expressed the location of juice consumption as a percentage of the respondents interviewed; B. Poll survey expressed the reason of juice consumption as a percentage of the respondents interviewed; Table S1. Description of juice samples composition, location of purchasing, and their benefits; Table S2. Antimicrobial resistance (%) of isolates selected from strawberries; Table S3. Antimicrobial resistance (%) of isolates selected from gooseberries; Table S4. Antimicrobial resistance (%) of isolates selected from juices.
