# Supplementary material for: Pathogenic Microorganisms Linked to Fresh Fruits and Juices Purchased at Low-Cost Markets in Ecuador, Potential Carriers of Antibiotic Resistance
Source: Antibiotics (Basel). 2023 Jan 22;12(2):236. doi: 10.3390/antibiotics12020236 (PMC9952111; doi:10.3390/antibiotics12020236)

**Figure S1.** A. Poll survey expressed the location of juice consumption as a percentage of the respondents interviewed; B. Poll survey expressed the reason of juice consumption as a percentage of the respondents interviewed.

1.
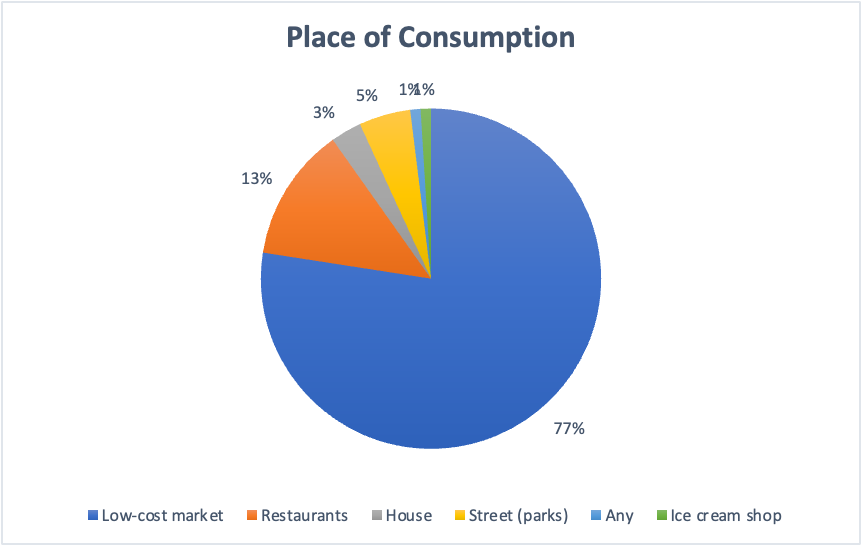

2.
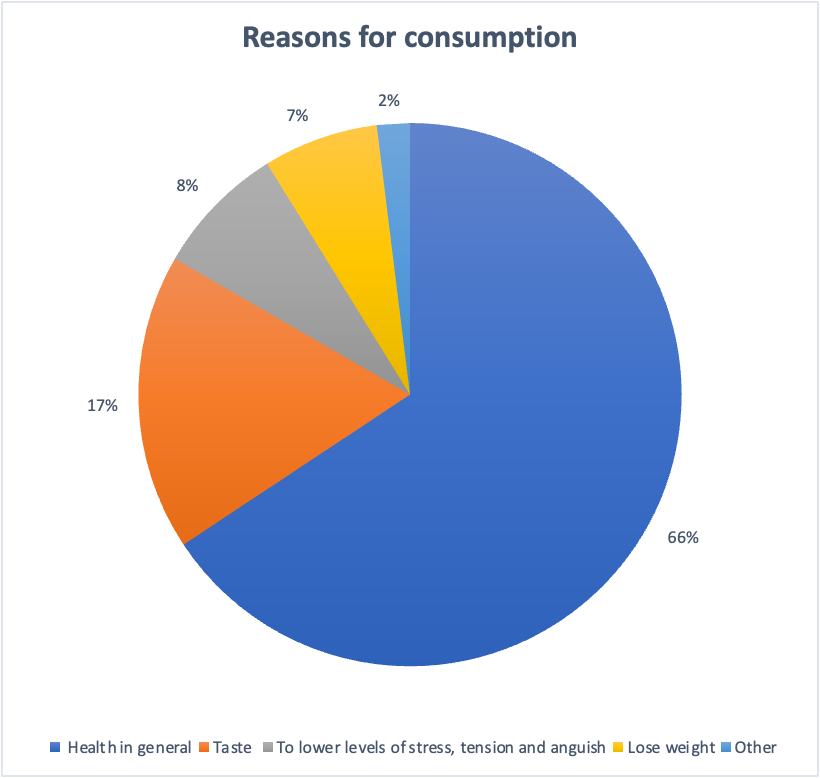

Supplement: Supplementary file 1 [file antibiotics-12-00236-s001.zip › Figure S1.docx]
