# Supplementary material for: Pathogenic Microorganisms Linked to Fresh Fruits and Juices Purchased at Low-Cost Markets in Ecuador, Potential Carriers of Antibiotic Resistance
Source: Antibiotics (Basel). 2023 Jan 22;12(2):236. doi: 10.3390/antibiotics12020236 (PMC9952111; doi:10.3390/antibiotics12020236)
Supplement: Supplementary file 1 [file antibiotics-12-00236-s001.zip › Table S1.docx]

**Table S1.** Description of juice samples composition, location of purchasing and their benefits.

| **Juice code** | **Sampling date** | **Selling location** | **Composition*** | **Health benefit** |  |
| --- | --- | --- | --- | --- | --- |
|  |  |  |  |  |  |
| B1 | 06/26/19 | Low-cost market Amazonas | Tree tomato, beetroot, guava | Blood circulation |  |
| B2 | 06/26/19 | Low-cost market Amazonas | Orange, alfalfa, gooseberry | Anemia |  |
| B3 | 06/26/19 | Low-cost market Amazonas | Orange, carrot | Eyes |  |
| B4 | 09/09/19 | Low-cost market Amazonas | Tree tomato | Aging |  |
| B5 | 09/09/19 | Low-cost market Amazonas | Blackberry, gooseberry | Anemia, immune system |  |
| B6 | 09/09/19 | Low-cost market Amazonas | Soursop (Guanábana) pulp | Cancer prevention |  |
| B7 | 11/25/19 | Park Centrica | Orange, strawberries, and papaya | Skin health |  |
|  |  |  |  |  |  |
| B8 | 11/25/19 | Low-cost market Santo Domingo | Carrot, apple, and celery | High pressure control |  |
|  |  |  |  |  |  |
| B9 | 11/25/19 | Low-cost market Santo Domingo | Blackberry, alfalfa, chicken liver | Anemia |  |
|  |  |  |  |  |  |
| B10 | 11/25/19 | Park Centrica | Grapefruit and oatmeal | Cholesterol, lose weight |  |
|  |  |  |  |  |  |
| B11 | 02/03/20 | Park Centrica | Pineapple, cucumber, orange, apple, and aloe vera | Colon health |  |
|  |  |  |  |  |  |
| B12 | 02/03/20 | Park Centrica | Carrot, spinach, milk, butter, and egg | Skin health |  |
|  |  |  |  |  |  |
| B13 | 02/03/20 | Park Centrica | Cucumber, apple, ginger, and celery | Headache |  |
|  |  |  |  |  |  |
| B14 | 02/03/20 | Park Centrica | Orange, papaya, grapes, and carrot | Heart health |  |
|  |  |  |  |  |  |
| B15 | 02/03/20 | Park Centrica | Orange, spinach, and cucumber | Bad breath |  |
|  |  |  |  |  |  |
| B16 | 02/03/20 | Park Centrica | Spinach, carrot, and apple | Depression |  |
|  |  |  |  |  |  |
| B17 | 07/21/20 | Low-cost market Santo Domingo | Papaya, apple, aloe vera and honey | Pimples |  |
|  |  |  |  |  |  |
| B18 | 07/21/20 | Low-cost market Santo Domingo | Ponimalta and egg | Increasing muscle mass |  |
|  |  |  |  |  |  |
| B19 | 07/21/20 | Low-cost market Santo Domingo | Orange, ginger, and honey | Cold of fly |  |
|  |  |  |  |  |  |
| B20 | 07/21/20 | Low-cost market Santo Domingo | Borojo (*Alibertia patinoi* fruits) and milk | Prevention of cardiocerebrovascular diseases, Aphrodisiac |  |
|  |  |  |  |  |  |
| * The composition was provided by the retailer. | | |  |  |  |
