# Supplementary material for: Pathogenic Microorganisms Linked to Fresh Fruits and Juices Purchased at Low-Cost Markets in Ecuador, Potential Carriers of Antibiotic Resistance
Source: Antibiotics (Basel). 2023 Jan 22;12(2):236. doi: 10.3390/antibiotics12020236 (PMC9952111; doi:10.3390/antibiotics12020236)
Supplement: Supplementary file 1 [file antibiotics-12-00236-s001.zip › Table S2.docx]

**Table S2.** Antimicrobial resistance (%) of isolates selected from strawberries.

| **Samples** | **Selected isolates** | **Antibiotic class** | | | | | | | |
| --- | --- | --- | --- | --- | --- | --- | --- | --- | --- |
|  |  | **Aminoglycosides** | | **Beta-lactamase inhibitors** | **Tetra-ciclyne** | **Cephalo-sporins** | **Glyco-peptide** | **Penicillin-like** | |
|  |  | **K30** | **CN10** | **AN10** | **TE30** | **CXM30** | **VAN30** | **AX25** | **MET5** |
| **SFS** | *E. coli* (n=30) | 81% | 14% | 53% | 69% | 14% | 100% | 47% | ND |
|  | *Shigella* spp. (n=20) | 25% | 75% | 0% | 0% | 100% | 0% | 25% | ND |
|  | *Enterobacter* spp. (n=28) | 16% | 33% | 22% | 13% | 9% | 83% | 38% | ND |
|  | *Staphylococcus* spp. (n=20) | 12% | 0% | 87% | 13% | 0% | 100% | 38% | 100% |
| **SFM** | *E. coli* (n=30) | 0% | 0% | 33% | 0% | 100% | 100% | 0% | ND |
|  | *Shigella* spp. (n=15) | 40% | 40% | 20% | 40% | 40% | 0% | 20% | ND |
|  | *Enterobacter* spp. (n=29) | 25% | 27% | 75% | 17% | 17% | 79% | 75% | ND |
|  | *Staphylococcus* spp. (n=20) | 45% | 44% | 78% | 0% | 25% | 100% | 67% | 100% |

% was calculated as no. total of indicator bacteria resistant / no. total isolates. Legend: SFS-strawberries from a local farm grower; SFM: strawberries from low-cost market; K30: kanamycin 30 (μg); CN10: gentamycin 10 (μg); AN10: ampicillin 10 (μg); AX25: amoxicillin 25 (μg); TE30: tetracycline 30 (μg); CXM: cefuroxime 30 (μg); VAN30: vancomycin 30 (μg); MET5: methicillin 5 (μg); ND: not determined.
