# Supplementary material for: Pathogenic Microorganisms Linked to Fresh Fruits and Juices Purchased at Low-Cost Markets in Ecuador, Potential Carriers of Antibiotic Resistance
Source: Antibiotics (Basel). 2023 Jan 22;12(2):236. doi: 10.3390/antibiotics12020236 (PMC9952111; doi:10.3390/antibiotics12020236)
Supplement: Supplementary file 1 [file antibiotics-12-00236-s001.zip › Table S3.docx]

**Table S3.** Antimicrobial resistance (%) of isolates selected from gooseberries.

| **Samples** | **Selected isolates** | **Antibiotic class** | | | | | | | |
| --- | --- | --- | --- | --- | --- | --- | --- | --- | --- |
|  |  | **Aminoglycosides** | **Beta-lactamase inhibitors** | | **Tetra-ciclyne** | **Cephalo-sporins** | **Glyco-peptide** | **Penicillin -like** | |
|  |  | **K30** | **CN10** | **AN10** | **TE30** | **CXM30** | **VAN30** | **AX25** | **MET5** |
| **PFS** | *Enterobacter* (n=20) | 30% | 13% | 60% | 0% | 35% | ND | 50% | ND |
|  | *Staphylococcus* spp. *(n=20)* | 0% | 9% | 65% | 0% | 26% | 71% | 52% | 100% |
| **PFM** | *Enterobacter* (n=20) | 25% | 38% | 75% | 13% | 25% | ND | 63% | ND |
|  | *Staphylococcus* spp. (n=20) | 14% | 29% | 43% | 0% | 25% | 60% | 29% | 100% |

% was calculated as no. total indicator bacteria resistant / no. total isolates. Legend: PFS: gooseberries form a local farm grower; PFM: gooseberries from low-cost market; K30: Kanamycin 30 (μg); CN10: Gentamycin 10 (μg); AN10: Ampicillin 10 (μg); AX25: Amoxicillin 25 (μg); TE30: Tetracycline 30 (μg); CXM: Cefuroxime 30 (μg); VAN30: Vancomycin 30 (μg); MET5: methicillin 5 (μg); ND: not determined.
