# Supplementary material for: Pathogenic Microorganisms Linked to Fresh Fruits and Juices Purchased at Low-Cost Markets in Ecuador, Potential Carriers of Antibiotic Resistance
Source: Antibiotics (Basel). 2023 Jan 22;12(2):236. doi: 10.3390/antibiotics12020236 (PMC9952111; doi:10.3390/antibiotics12020236)
Supplement: Supplementary file 1 [file antibiotics-12-00236-s001.zip › Table S4.docx]

**Table S4.** Antimicrobial resistance (%) of isolates selected from juices.

| **Samples** | **Selected isolates** | **Antibiotic class (%** | | | | | | |
| --- | --- | --- | --- | --- | --- | --- | --- | --- |
|  |  | **Aminoglycosides** | | **Beta-lactamase inhibitors** | **Tetra-ciclyne** | **Cephalon-sporin** | **Penicillin-like** | |
|  |  | **K30** | **CN10** | **AN10** | **TE30** | **CXM30** | **AMC30** | **AX25** |
| **B1** | *Shigella* spp. (n=20) | 50% | 35% | 35% | 50% | 25% | 30% | 100% |
|  | *Enterobacter* spp. (n=22) | 68% | 42% | 33% | 27% | 50% | 55% | 33% |
| **B2** | *E. coli* (n=12) | 33% | 67% | 41% | 67% | 67% | 50% | 64% |
|  | *Shigella* spp. (n=10) | 30% | 50% | 30% | 70% | 60% | 30% | 60% |
|  | *Enterobacter* (n=10) | 70% | 80% | 50% | 70% | 60% | 30% | 70% |
| **B3** | *Salmonella* spp. (n=10) | 10% | 90% | 90% | 90% | 90% | 90% | 90% |
|  | *Shigella* spp. (n=10) | 90% | 80% | 60% | 80% | 70% | 90% | 100% |
|  | *Enterobacter* (n=10) | 100% | 80% | 30% | 0% | 10% | 10% | 90% |
| **B4** | *Salmonella* spp. (n=10) | 30% | 30% | 60% | 40% | 100% | 20% | 100% |
|  | *Shigella* spp. (n=10) | 20% | 30% | 50% | 20% | 0% | 20% | 50% |
|  | *Enterobacter* spp. (n=10) | 90% | 90% | 90% | 20% | 80% | 80% | 70% |
|  | *E. coli* (n=12) | 25% | 58% | 67% | 10% | 25% | 67% | 92% |
| **B5** | *Enterobacter* spp. (n=12) | 25% | 92% | 58% | 42% | 67% | 67% | 100% |
|  | *Shigella* spp. (n=10) | 20% | 30% | 50% | 20% | 0% | 70% | 100% |
|  | *Salmonella* spp. (n=10) | 40% | 100% | 40% | 40% | 0% | 20% | 100% |
| **B6** | *Salmonella* spp. (n=13) | 62% | 85% | 62% | 23% | 62% | 46% | 100% |
|  | *Enterobacter* spp. (n=29) | 48% | 24% | 41% | 14% | 28% | 59% | 100% |
| **B7** | *Salmonella* spp. (n=10) | 80% | 50% | 80% | 30% | 100% | 100% | 90% |
|  | *Shigella* spp. (n=10) | 80% | 50% | 80% | 30% | 100% | 100% | 100% |
|  | *Enterobacter* spp. (n=10) | 80% | 50% | 80% | 30% | 100% | 100% | 100% |
|  | *E. coli* (n=11) | 73% | 45% | 73% | 27% | 91% | 91% | 100% |
| **B8** | *Salmonella* spp. (n=10) | 80% | 50% | 80% | 30% | 100% | 100% | 100% |
|  | *Shigella* spp. (n=10) | 80% | 50% | 80% | 30% | 100% | 100% | 100% |
|  | *Enterobacter* spp. (n=10) | 80% | 50% | 80% | 20% | 100% | 100% | 100% |
| **B9** | *Shigella* spp. (n=16) | 13% | 100% | 94% | 81% | 69% | 25% | 100% |
|  | *Enterobacter* spp. (n=23) | 35% | 35% | 52% | 83% | 100% | 17% | 100% |
| **B10** | *Salmonella* spp. (n=10) | 80% | 20% | 80% | 20% | 100% | 100% | 100% |
|  | *Shigella* spp. (n=10) | 40% | 30% | 90% | 20% | 100% | 100% | 100% |
|  | *Enterobacter* (n=10) | 80% | 20% | 80% | 20% | 90% | 80% | 100% |
|  | *E. coli* (n=12) | 100% | 100% | 100% | 17% | 100% | 100% | 100% |
| **B11** | *Salmonella* spp. (n=10) | 80% | 80% | 80% | 20% | 90% | 80% | 100% |
|  | *Shigella* spp. (n=10) | 80% | 20% | 40% | 20% | 40% | 80% | 100% |
|  | *Enterobacter* spp. (n=10) | 80% | 30% | 80% | 30% | 90% | 80% | 100% |
|  | *E. coli* (n=11) | 100% | 100% | 100% | 100% | 100% | 100% | 100% |
| **B12** | *Salmonella* spp. (n=16) | 69% | 50% | 100% | 69% | 100% | 100% | 100% |
|  | *Enterobacter* spp. (n=20) | 100% | 100% | 100% | 55% | 100% | 100% | 100% |
| **B13** | *Salmonella* spp. (n=10) | 100% | 100% | 100% | 20% | 90% | 100% | 100% |
|  | *Shigella* spp. (n=10) | 80% | 10% | 100% | 20% | 80% | 100% | 100% |
|  | *Enterobacter* (n=10) | 100% | 100% | 100% | 100% | 90% | 100% | 100% |
| **B14** | *Salmonella* spp. (n=10) | 100% | 0% | 100% | 0% | 90% | 100% | 100% |
|  | *Shigella* spp. (n=10) | 100% | 100% | 100% | 100% | 90% | 100% | 100% |
|  | *Enterobacter* spp. (n=19) | 89% | 100% | 100% | 100% | 100% | 100% | 100% |
| **B15** | *Salmonella* spp. (n=10) | 100% | 20% | 100% | 20% | 20% | 100% | 100% |
|  | *Shigella* spp. (n=10) | 100% | 0% | 100% | 100% | 90% | 100% | 100% |
|  | *E. coli* (n=12) | 100% | 100% | 100% | 100% | 100% | 100% | 100% |
| **B16** | *Salmonella* spp. (n=10) | 100% | 60% | 100% | 33% | 100% | 100% | 100% |
|  | *Shigella* spp. (n=10) | 100% | 60% | 100% | 50% | 100% | 100% | 100% |
|  | *Enterobacter* spp. (n=10) | 100% | 100% | 100% | 30% | 100% | 100% | 100% |
|  | *E. coli* (n=12) | 0% | 0% | 0% | 0% | 0% | 0% | 100% |
| **B17** | *Salmonella* spp. (n=10) | 100% | 50% | 100% | 50% | 100% | 100% | 100% |
|  | *Shigella* spp. (n=10) | 80% | 50% | 100% | 40% | 100% | 100% | 100% |
|  | *Enterobacter* spp. (n=10) | 80% | 100% | 100% | 20% | 100% | 100% | 100% |
|  | *E. coli* (n=10) | 56% | 30% | 100% | 100% | 100% | 100% | 100% |
| **B18** | *E. coli* (n=16) | 100% | 56% | 100% | 100% | 100% | 100% | 100% |
|  | *Shigella* spp. (n=18) | 94% | 11% | 100% | 56% | 100% | 100% | 100% |
| **B19** | *Enterobacter* spp. (n=10) | 100% | 100% | 100% | 100% | 100% | 100% | 100% |
|  | *Salmonella* spp. (n=10) | 100% | 70% | 100% | 100% | 100% | 100% | 100% |
|  | *E. coli* (n=16) | 100% | 63% | 100% | 100% | 81% | 100% | 100% |
| **B20** | *Shigella* spp. (n=10) | 60% | 10% | 100% | 0% | 100% | 100% | 100% |
|  | *E. coli* (n=10) | 70% | 10% | 100% | 100% | 100% | 100% | 100% |

% was calculated as no. total indicator bacteria resistant / no. total isolates. Legend: K30: Kanamycin 30 (μg); CN10: Gentamycin 10 (μg); AN10: Ampicillin 10 (μg); AX25: Amoxicillin 25 (μg); TE30: Tetracycline 30 (μg); CXM30: Cefuroxime 30 (μg); AMC30: amoxicillin: clavulanic acid (20/10 μg).
